# Clinical observation of diminished bone quality and quantity through longitudinal HR-pQCT-derived remodeling and mechanoregulation

**DOI:** 10.1038/s41598-022-22678-z

**Published:** 2022-10-26

**Authors:** Caitlyn J. Collins, Penny R. Atkins, Nicholas Ohs, Michael Blauth, Kurt Lippuner, Ralph Müller

**Affiliations:** 1 Institute for Biomechanics, ETH Zurich, Zurich, Switzerland; 2 Department of Biomedical Engineering and Mechanics, Virginia Tech, Blacksburg, VA USA; 3 Department of Osteoporosis, Bern University Hospital, University of Bern, Bern, Switzerland; 4 Scientific Computing and Imaging Institute, University of Utah, Salt Lake City, UT USA; 5 Department of Orthopedics and Trauma Surgery, Medical University of Innsbruck, Innsbruck, Austria; 6 Clinical Medical Department DePuy Synthes, Zuchwil, Switzerland

**Keywords:** Translational research, Time-lapse imaging, X-ray tomography, Musculoskeletal models, Image processing

## Abstract

High resolution peripheral quantitative computed tomography (HR-pQCT) provides methods for quantifying volumetric bone mineral density and microarchitecture necessary for early diagnosis of bone disease. When combined with a longitudinal imaging protocol and finite element analysis, HR-pQCT can be used to assess bone formation and resorption (i.e., remodeling) and the relationship between this remodeling and mechanical loading (i.e., mechanoregulation) at the tissue level. Herein, 25 patients with a contralateral distal radius fracture were imaged with HR-pQCT at baseline and 9–12 months follow-up: 16 patients were prescribed vitamin D3 with/without calcium supplement based on a blood biomarker measures of bone metabolism and dual-energy X-ray absorptiometry image-based measures of normative bone quantity which indicated diminishing (n = 9) or poor (n = 7) bone quantity and 9 were not. To evaluate the sensitivity of this imaging protocol to microstructural changes, HR-pQCT images were registered for quantification of bone remodeling and image-based micro-finite element analysis was then used to predict local bone strains and derive rules for mechanoregulation. Remodeling volume fractions were predicted by both average values of trabecular and cortical thickness and bone mineral density (R^2^ > 0.8), whereas mechanoregulation was affected by dominance of the arm and group classification (*p* < 0.05). Overall, longitudinal, extended HR-pQCT analysis enabled the identification of changes in bone quantity and quality too subtle for traditional measures.

## Introduction

Approximately 10% of older adults suffer from osteoporosis and another 40% of the same population is affected by osteopenia^1,2^. Both conditions are characterized by low bone mass and a high risk of debilitating and often life-threatening fractures. In fact, the lifetime probability of a major osteoporotic fracture caused by poor bone health (i.e. hip, spine, proximal humerus, or distal radius) is 20% in men and 50% in women^3,4^. However, patients with osteopenia are often left undiagnosed and untreated due to a more subtle deterioration of bone quality and quantity^5,6^, leaving them susceptible to further bone degeneration. When recognized clinically, patients with low bone mass are often initially recommended supplements, such as vitamin D3 or calcium. If bone mass is not increased or sustained, patients may be prescribed osteoanabolic or anti-resorptive drugs; however, these treatments are not always effective and often have diminishing efficacy with time resulting in poor outcomes long-term.

Aside from the issues with potential treatments, a major barrier in helping patients with either osteoporosis or osteopenia is the lack of preventative screening. Clinically, bone quantity is often measured through quantification of bone mineral content (BMC, in grams) and areal bone mineral density (BMD, in g/cm^2^) of the radius, hip, and/or spine using dual-energy X-ray absorptiometry (DXA). With the creation of large normative and longitudinally measured databases, the use of DXA and BMD measurements has become the standard of care in the clinical diagnosis and management of osteoporosis and osteopenia. Here, diagnostic thresholds, established as standard deviations above or below a young adult reference mean (T-scores), are used to categorize patients into descriptive categories: normal (T-score ≥ − 1 SD), low bone mass or osteopenia (T-score < − 1 and > − 2.5 SD), and osteoporosis (T-score ≤ − 2.5 SD)^7^. However, the first clinically recognized sign of low bone mass is often a fragility fracture and measurements of BMD by DXA may not be prescribed until after a fracture^8^. Further, BMD measurements from DXA have been shown to lack the necessary sensitivity to serve as an effective fracture risk assessment tool^9–11^, even in combination with individual patient risk factors in the Fracture Risk Assessment Tool (FRAX®)^12,13^. The lack of sensitivity of DXA, with or without the addition of FRAX, suggests that bone quality and microarchitecture play a key role in the prediction of individual fracture risk.

To better account for more subtle changes in bone quality and strength, indicative of the onset of osteopenia or osteoporosis, alternative image-based metrics have been introduced to assess bone health and fracture risk. Trabecular Bone Score (TBS)^14^, a DXA-based tool for approximating bone microstructure using texture-based analysis, has been shown to increase the prognostic value of BMD and FRAX^15,16^. However, this technique only provides a 2D assessment of the lumbar spine and has been shown to have lower reproducibility than DXA^14^. Biomechanical computed tomography (CT) analysis (BCT), an image-based finite element (FE) analysis designed to measure bone strength from clinical CT images of the hip or spine, has recently been approved for osteoporosis diagnostic testing in the United States^17^. Taking into account both 3D patient bone geometry and material properties, BCT has been used to successfully group patients into low and high fracture risk categories and provide a comprehensive measure of bone strength^17,18^. Although BCT seems to be a powerful tool for assessing bone health, varied implementations across groups and institutions result in different bone strength predictions. Due to this variability, the assessment of longitudinal changes using BCT is more robust than absolute measures^19^. However, the widespread use of longitudinal BCT is unlikely since the relatively high radiation dose of each scan (286–506 µSV for a low-dose hip CT) limits BCT to the use of scans acquired for unrelated clinical purposes^20^.

High-resolution peripheral quantitative CT (HR-pQCT), an emerging diagnostic imaging technology with low effective radiation dose (3–5 µSv), enables the assessment of 3D bone morphometrics and densitometrics, including volumetric BMD^21^, at peripheral sites such as the distal radius and tibia. In addition to direct measurement of BMD in 3D, these high-resolution images can be used to evaluate both compartment specific (i.e. cortical and trabecular) structural properties and bone mechanical properties, such as stiffness and strength through FE analysis^22,23^. A study including international patient cohorts found HR-pQCT-based estimated failure load at the tibia and radius to be the strongest predictors of incident fracture, independent of femoral neck DXA-based BMD and FRAX^6^. Further, the ability to track changes in the cortical and trabecular compartments has revealed both age-related^24–26^ and disease-specific characteristics^6,26–28^ not previously realized using regional DXA-based measures. However, the regional and tissue-level measures do not fully capitalize on the capabilities of HR-pQCT, which when combined with a longitudinal imaging protocol include microstructural analysis of bone remodeling^29^ through dynamic morphometry which allows for the direct quantification of bone formation and resorption and of the association of this remodeling with the mechanical loading of the bone, i.e. mechanoregulation^30,31^. To date, the ability of such longitudinal, extended HR-pQCT analysis tools to detect clinically relevant changes in bone quality and quantity has yet to be thoroughly investigated.

The purpose of this study was to investigate the application of longitudinal HR-pQCT imaging and associated remodeling and mechanoregulation analyses in the radius of human subjects. We hypothesized that these longitudinal, extended analyses would provide increased sensitivity to the assessment bone microarchitecture and mineral density (i.e. quality and quantity) relative to traditional clinical methods. For this analysis, we utilized longitudinal HR-pQCT images of patients in three groups, those with normal bone mass and those with low bone mass, who were either prescribed no supplements or vitamin D3 with/without calcium supplements, to understand whether high-resolution 3D imaging would be useful in the clinical diagnosis and long-term management of bone health. These methods could provide the means to more accurately assess changes in bone microarchitecture for patients at risk of osteopenia and osteoporosis, overcoming the current limitations of existing clinical assessment techniques.

## Results

Of the 25 subjects, nine were not prescribed any form of additional treatment (NoSupp) while 16 were prescribed supplements (i.e., vitamin D3 with/without calcium), based on low values of blood-based bone markers at baseline (calcium, 25-hydroxyvitamin D [25(OH)D], and parathyroid hormone [PTH]). Of the 16 subjects who were prescribed some form of supplement, nine had no femur, lumbar spine, or radius T-Scores below − 2.5 (LowSupp) and seven had at least one of these T-Scores at or below − 2.5 (OPSupp) (Table 1). After adjusting for baseline values, neither imaging interval nor age had a statistically significant effect on post-intervention values. Therefore, these were excluded from any further analysis.

**Table 1:** Patient demographics for subject groups.

Metric	NoSupp (N = 9)	LowSupp (N = 9)	OPSupp (N = 7)	*p* val
Sex	6 Female (2/4) 3 Male	6 Female (3/3) 3 Male	6 Female (2/4) 1 Male	0.647
Dominance of the Evaluated Arm	4 Dominant 3 Non-Dominant 2 Ambidextrous	4 Dominant 5 Non-Dominant	1 Dominant 5 Non-Dominant 1 Ambidextrous	0.310
Fracture Mechanism	5 Low Impact 4 High Impact	8 Low Impact 1 High Impact	4 Low Impact 3 High Impact	0.258
Supplement	–	3 Vitamin D 6 Combined	2 Vitamin D 5 Combined	0.958
Age (years)	56 ± 17	53 ± 12	58 ± 19	0.564
BMI (kg/m^2^)	22.5 ± 3.5	25.3 ± 3.7	23.5 ± 2.7	0.368
Supplement Interval (months)	–	10.5 (9.2–11.5)	10.5 (10.3–11.1)	0.957
Imaging Interval (months)	11.2 (8.7–12.1)	10.8 (9.2–11.7)	11.1 (10.3–11.3)	0.788
Fracture to Baseline (months)	0.7 (0.3–3.1)	0.7 (0.3–1.4)	0.8 (0.3–1.2)	0.838

Supplement interval represents the duration of supplementation prior to the post-intervention imaging session. Imaging interval represents the time between baseline and post-intervention imaging. Whether participants were pre- or post-menopausal is indicated after the number of females in the format of (pre/post). Data presented as either mean ± standard deviation or median (range). BMI, body mass index; N, number per group; Combined, vitamin D3 and calcium.

### Static Morphometry and Densitometry

Subjects from the three groups had varied radius and lumbar spine T-scores as well as cortical bone mineral density (Ct.BMD) values but did not differ in other measures of bone quality or quantity (Table 2). Group, dominance of the evaluated arm, and sex were investigated covariates in the ANCOVA. Group had a near significant impact on post-intervention adjusted mean trabecular thickness (Tb.Th) (Table 3). Here, NoSupp (0.223 ± 0.001) had significantly higher adjusted mean Tb.Th than LowSupp (0.218 ± 0.002, *p* = 0.038, 2.3% reduction) but not OPSupp (0.221 ± 0.002, *p* = 0.68, 0.9% reduction). Similarly, sex had a significant impact on adjusted mean cortical thickness (Ct.Th) (Table 3), where males had significantly higher Ct.Th (0.946 ± 0.048) in comparison to females (0.810 ± 0.034, *p* = 0.025, 15.5% reduction). Dominance of the evaluated arm had no significant impact on post-intervention values for any of the static morphometric parameters.

**Table 2:** Averaged patient measures of bone morphometrics, densitometrics, remodeling volume fractions, and mechanical parameters.

	Metric (Unit)	NoSupp (N = 9)	LowSupp (N = 9)	OPSupp (N = 7)	*p* val
DXA Bone Density	Radius T-Score	− 1.2 ± 1.0	− 1.4 ± 0.1	− 2.8 ± 1.1	0.005 ^
	Femur T-Score	− 0.7 ± 1.0	− 0.7 ± − 0.6	− 1.5 ± 0.5	0.124
	Spine T-Score	− 0.6 ± 1.3	− 1.3 ± 0.9	− 2.1 ± 0.8	0.045 ^
HR-pQCT Microarchitecture	Tt.BMD (mg HA/cm^3^)	271.3 (38.4)	263.1 (68.8)	205.4 (72.1)	0.071
	Tb.BMD (mg HA/cm^3^)	130.8 (50.7)	129.8 (46.0)	105.8 (34.0)	0.559
	Ct.BMD (mg HA/cm^3^)	860.8 (71.9)	896.4 (35.5)	790.0 (127.9)	0.021°
	BV/TV (%)	0.190 (0.076)	0.189 (0.073)	0.163 (0.044)	0.569
	Tb.N (mm^−1^)	1.280 (0.220)	1.342 (0.412)	1.282 (0.250)	0.772
	Tb.Sp (mm)	0.748 (0.129)	0.729 (0.254)	0.766 (0.174)	0.701
	Tb.Th (mm)	0.228 (0.030)	0.218 (0.012)	0.217 (0.013)	0.444
	Tb.Sp (mm)	0.748 (0.129)	0.729 (0.254)	0.766 (0.174)	0.701
	Ct.Th (mm)	0.961 (0.119)	0.926 (0.131)	0.657 (0.209)	0.078
	Ct.Po (%)	0.008 (0.004)	0.005 (0.002)	0.006 (0.004)	0.111
	Ct.Po.Dm (mm)	0.192 (0.043)	0.178 (0.039)	0.171 (0.018)	0.234
Remodeling Volume Fractions	Tb F (mm^3^/mm^3^)	0.340 (0.165)	0.297 (0.148)	0.365 (0.197)	0.001 *°
	Tb Rs (mm^3^/mm^3^)	0.365 (0.218)	0.356 (0.205)	0.358 (0.173)	0.900
	Ct F (mm^3^/mm^3^)	0.044 (0.061)	0.034 (0.050)	0.068 (0.085)	0.000 ^°
	Ct Rs (mm^3^/mm^3^)	0.060 (0.060)	0.045 (0.054)	0.080 (0.095)	0.000 ^°
Mechanics	Stiffness (kN/mm)	52.3 (41.5)	71.5 (18.0)	47.8 (27.0)	0.102
	Tb 5th Eff Strain (µε)	628 (299)	722 (387)	690 (271)	0.788
	Tb 10th Eff Strain (µε)	1020 (272)	1200 (610)	1050 (359)	0.753
	Tb 25th Eff Strain (µε)	2140 (445)	2500 (1080)	1980 (479)	0.407
	Tb 50th Eff Strain (µε)	4350 (1100)	4980 (1660)	3950 (675)	0.305
	Tb 75th Eff Strain (µε)	8250 ± 1560	8410 ± 989	7330 ± 817	0.210
	Ct 5th Eff Strain (µε)	1140 (345)	1760 (381)	1330 (280)	0.178
	Ct 10th Eff Strain (µε)	1920 (516)	2270 (422)	1790 (274)	0.093
	Ct 25th Eff Strain (µε)	3100 ± 1000	3350 ± 534	2670 ± 410	0.232
	Ct 50th Eff Strain (µε)	4730 ± 969	5130 ± 692	4370 ± 546	0.198
	Ct 75th Eff Strain (µε)	7160 ± 739	7440 ± 697	6900 ± 675	0.382

Data presented as either mean ± standard deviation or median (interquartile range). DXA measurements were acquired three-weeks post-fracture, HR-pQCT microarchitecture measures were averaged between baseline and post-intervention measures, and remodeling volume fractions were calculated over the imaging interval. The full-field effective strain data in each bone compartment was sampled at the 5th, 10th, 25th, 50th, and 75th percentiles to enable quantitative comparisons of the strain distribution across each finite element model. *p* values represent group differences as assessed by Kruskal–Wallis H test (HR-pQCT microarchitecture, remodeling volume fractions, and mechanics represented as median (IQR)) or one-way analysis of variance (DXA bone density and mechanics represented as mean ± standard deviation). Significant differences found from the post-hoc analysis are indicated as * between NoSupp and LowSupp, ^ between NoSupp and OPSupp, and ° between LowSupp and OPSupp from post-hoc analysis using Dunn's test with bonferroni correction for microarchitecture or Tukey–Kramer for T-Scores and remodeling volume fractions. A threshold of 320 mg/cm^3^ was used for remodeling volume fractions. N, number per group; BMD, bone mineral density; BV/TV, bone volume fraction; Tb, trabecular; N, number; Sp, separation; Th, thickness; Ct, cortical; Po, porosity; Po.Dm, cortical pore diameter; F, formation; Rs, resorption; Eff, effective strain, µε, microstrain.

**Table 3:** ANCOVA summary for static morphometric parameters.

Whole Bone				Cortical Bone				Trabecular Bone			
	df	F	*p* val		df	F	*p* val		df	F	*p* val
Tt.BMD				Ct.BMD				Tb.BMD			
Group	2	0.95	0.407	Group	2	0.85	0.443	Group	2	0.96	0.403
Sex	1	2.36	0.142	Sex	1	1.96	0.178	Sex	1	0.18	0.676
Arm	2	0.74	0.492	Arm	2	1.42	0.266	Arm	2	1.42	0.267
BV/TV				Ct.Th				Tb.N			
Group	2	1.42	0.268	Group	2	0.66	0.529	Group	2	0.31	0.735
Sex	1	0.13	0.724	Sex	1	5.42	0.032	Sex	1	0.72	0.408
Arm	2	1.00	0.388	Arm	2	1.02	0.380	Arm	2	1.48	0.253
				Ct.Po				Tb.Th			
				Group	2	0.16	0.850	Group	2	3.01	0.075
				Sex	1	0.89	0.359	Sex	1	0.03	0.875
				Arm	2	0.07	0.933	Arm	2	1.77	0.199
				Ct.Po.Dm				Tb.Sp			
				Group	2	0.01	0.986	Group	2	0.17	0.850
				Sex	1	2.43	0.136	Sex	1	0.01	0.935
				Arm	2	0.16	0.850	Arm	2	1.89	0.179

*p* values represent contrast differences as assessed by Tukey’s HSD method. BMD, bone mineral density; BV/TV, bone volume fraction; Tb, trabecular; N, number; Sp, separation; Th, thickness; Ct, cortical; Po, porosity; Po.Dm, cortical pore diameter.

### Dynamic morphometry

**Figure 1:**
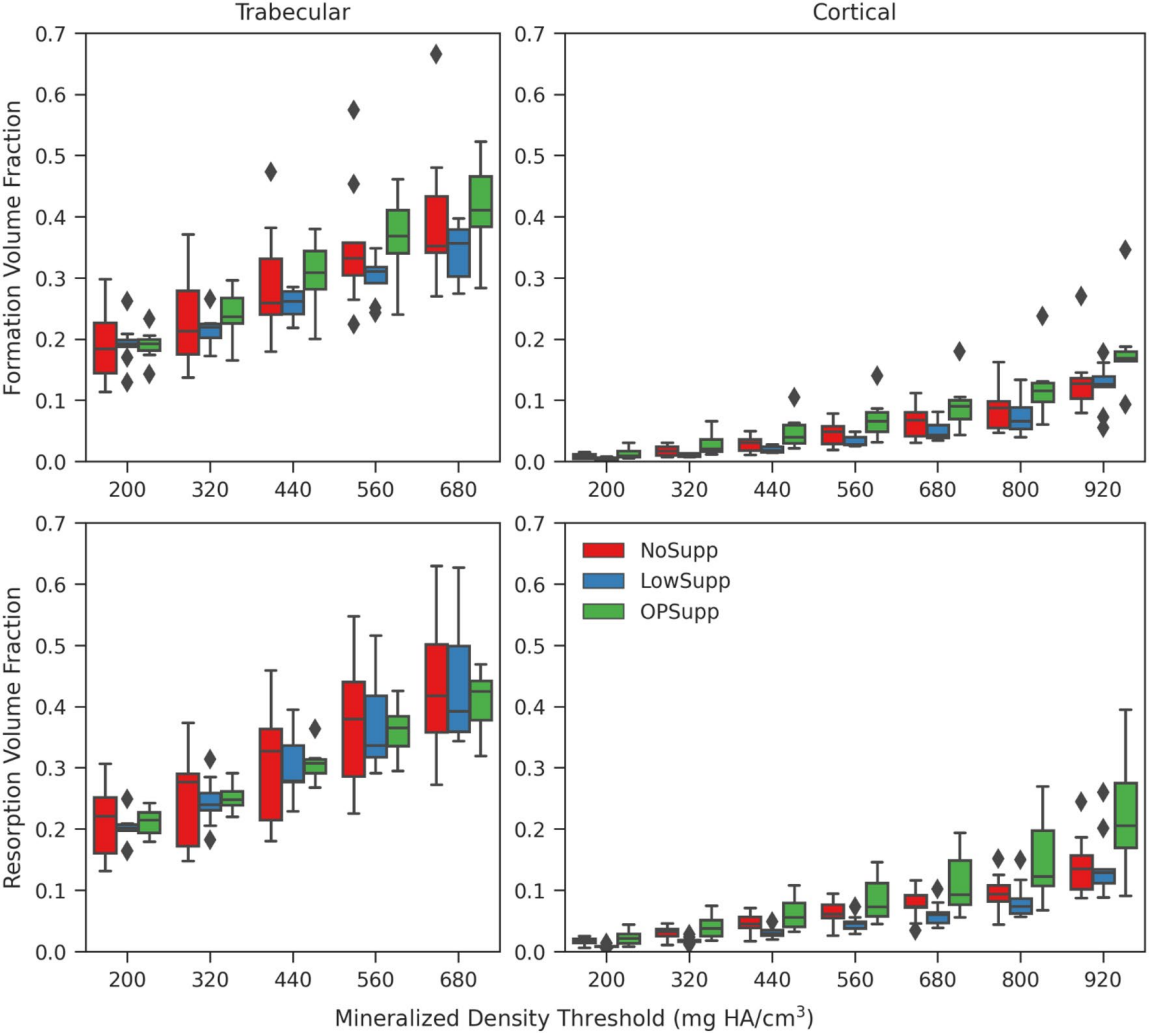
The formation and resorption volume fractions for each mineralized density threshold ranging from 200 mg Hydroxyapatite (HA)/cm^3^ to 680 mg HA/cm^3^ for trabecular bone and 920 mg HA/cm^3^ for cortical bone with 120 mg HA/cm^3^ intervals.

Formation and resorption volume fractions of the cortical and trabecular regions increased with mineralized bone density threshold (Fig. 1). Overall differences were observed among groups for trabecular formation and cortical formation and resorption using a standardized bone mineral density threshold of 320 mg/mm^3^ (Table 2); however, when considering all of the results, threshold-based differences were not significant for any of the volume fractions.

When evaluating against demographics and the averaged morphometric values, cortical formation volume fraction was predicted by cortical resorption, averaged Ct.BMD, and averaged Ct.Th (Table 4). Similarly, cortical resorption volume fraction was predicted by cortical formation, averaged Ct.BMD and averaged Ct.Th. Trabecular formation volume fraction was predicted by trabecular resorption, averaged total bone volume fraction (BV/TV), and averaged trabecular bone mineral density (Tb.BMD). Conversely, trabecular resorption volume fraction was predicted by trabecular formation and averaged Tb.Th.

**Table 4:** PLS regression summary for formation and resorption volume fractions.

	Variable	VIP	Regression coefficient
Cortical Formation	Intercept	–	6.726
	Ct.vBMD	1.057	− 0.008
	Cortical Resorption	1.051	0.008
	Ct.Th	0.882	0.003
	R^2^ = 0.937, Q^2^ = 0.892, RMSEP = 1.83		
Cortical Resorption	Intercept	–	2.957
	Cortical Formation	1.090	0.010
	Ct.vBMD	1.015	− 0.003
	Ct.Th	0.885	− 0.002
	R^2^ = 0.881, Q^2^ = 0.829, RMSEP = 3.98		
Trabecular Formation	Intercept	–	0.357
	Trabecular Resorption	1.291	0.046
	BV/TV	0.824	− 0.002
	Tb.vBMD	0.808	− 0.001
	R^2^ = 0.797, Q^2^ = 0.712, RMSEP = 0.001		
Trabecular Resorption	Intercept	–	0.242
	Trabecular Formation	1.165	0.042
	Tb.Th	0.801	− 0.010
	R^2^ = 0.822, Q^2^ = 0.764, RMSEP = 0.001		

VIP, variable influence on projection; Ct, cortical; vBMD, volumetric bone mineral density; Th, thickness; BV/TV, total bone volume fraction; Tb, trabecular; RMSEP, root mean squared error of prediction.

### Mechanics and mechanoregulation

Group, dominance of the evaluated arm, and sex were investigated as covariates to whole bone apparent compressive stiffness, cortical effective strain, and trabecular effective strain in the ANCOVA. Sex had no statistically significant effect on post-intervention values for any of the measured mechanics. None of the assessed covariates had an impact on post-intervention apparent stiffness. For post-intervention cortical and trabecular effective strain, group and dominance of the arm had a significant (*p* < 0.05) or near significant (*p* < 0.10) impact on post-intervention values.

**Figure 2:**
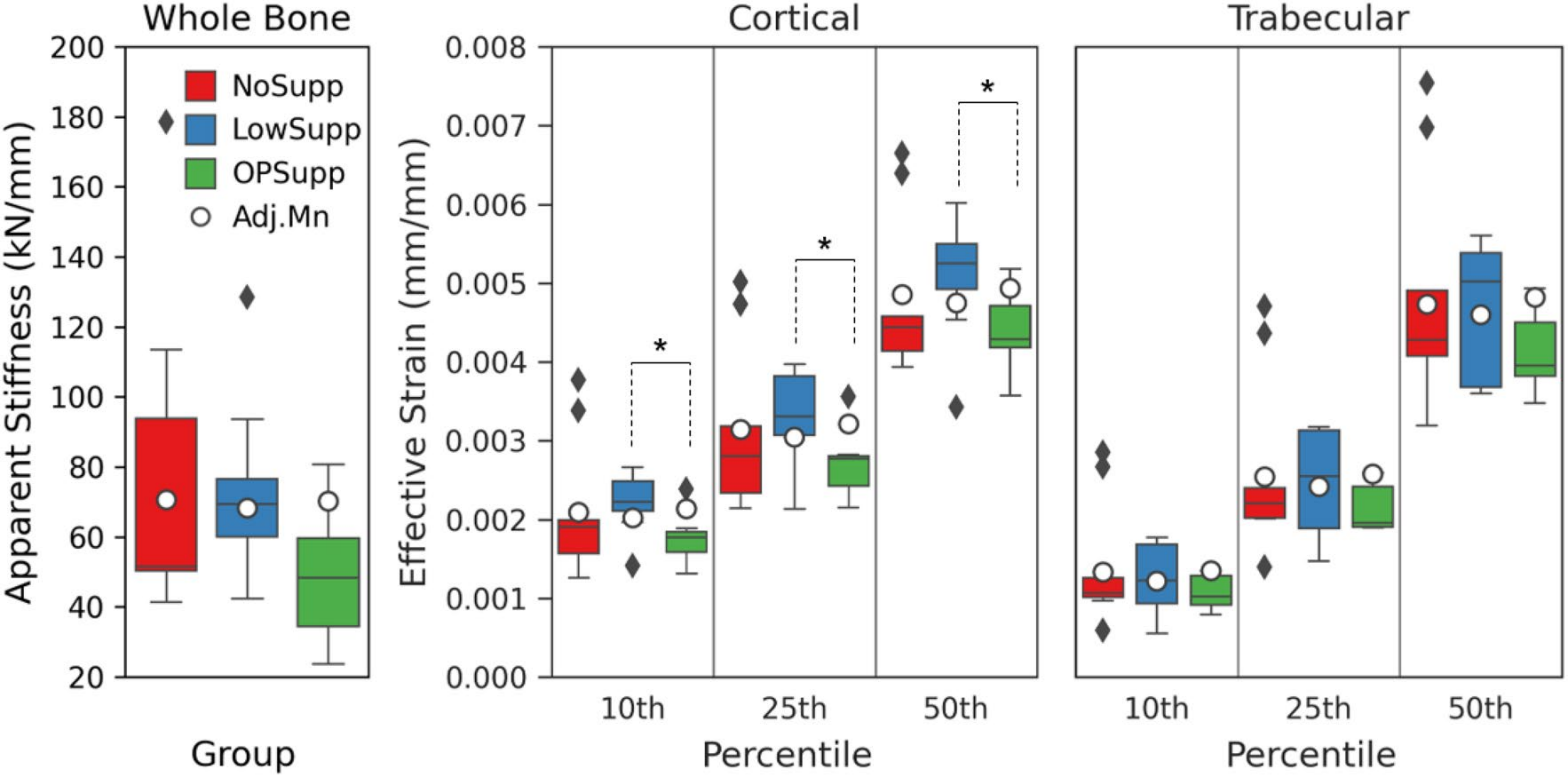
Post-intervention mechanical properties and adjusted means within the distal radius for NoSupp, LowSupp, and OPSupp. Whole bone apparent stiffness varied among groups, decreasing with treatment and worsening DXA scores; the lowest, post-intervention adjusted mean was detected in LowSupp (left). Cortical (middle) and trabecular (right) effective strain distributions, represented as discrete percentiles (10th, 25th, and 50th), reveal differences in post-intervention adjusted means between LowSupp and OPSupp within the cortex. (*) indicates significant contrasts between groups (*p* < 0.05).

Within the cortex, group had a significant impact on 10th and 25th percentile and near significant impact on 5th percentile and median adjusted effective strain (Table 5). In comparison to LowSupp, OPSupp had significantly higher cortical adjusted effective strain at the 10th percentile (LowSupp: 2020 ± 30 µɛ, OPSupp: 2140 ± 40 µɛ, *p* = 0.026), 25th percentile (LowSupp: 3050 ± 30 µɛ, OPSupp: 3210 ± 40 µɛ, *p* = 0.011), and median (LowSupp: 4750 ± 50 µɛ, OPSupp: 4940 ± 60 µɛ, *p* = 0.043) (Figure 2). No significant or near significant contrasts were detected among groups for the 5th or 75th percentile cortical adjusted effective strain. Arm had a significant impact on the 25th percentile and median cortical adjusted effective strain and near significant impact on 10th percentile cortical adjusted effective strain (Table 5). Ambidextrous (A) arms had significantly higher cortical adjusted effective strain than the non-dominant (ND) arms at the 10th percentile (A: 2170 ± 50 µɛ, ND: 2020 ± 30 µɛ, *p* = 0.046), 25th percentile (A: 3210 ± 40 µɛ, ND: 3050 ± 30 µɛ, *p* = 0.011) and median (A: 4940 ± 60 µɛ, ND: 4750 ± 50 µɛ, *p* = 0.043). For the 25th percentile cortical adjusted effective strain, ambidextrous arms were also nearly significantly higher than in the dominant (D) arms (A: 3210 ± 40 µɛ, D: 3090 ± 30 µɛ, *p* = 0.071).

**Table 5:** ANCOVA summary for mechanical parameters.

Whole Bone				Cortical Bone				Trabecular Bone		
	df	F	*p* val		df	F	*p* val	df	F	*p* val
Apparent Stiffness				5th Eff Strain						
Group	2	1.87	0.182	Group	2	3.51	*0.051*	2	4.40	**0.028**
Sex	1	0.97	0.399	Sex	1	1.41	0.250	1	3.39	*0.082*
Arm	2	2.11	0.164	Arm	2	2.44	0.116	2	0.79	0.468
				10th Eff Strain						
				Group	2	4.85	**0.021**	2	4.11	**0.034**
				Sex	1	2.52	0.130	1	3.43	*0.080*
				Arm	2	3.37	*0.057*	2	1.16	0.334
				25th Eff Strain						
				Group	2	5.94	**0.010**	2	3.20	*0.065*
				Sex	1	2.98	0.101	1	3.38	*0.082*
				Arm	2	5.01	**0.019**	2	2.15	0.145
				Median Eff Strain						
				Group	2	3.53	*0.051*	2	2.81	*0.087*
				Sex	1	1.46	0.243	1	3.20	*0.090*
				Arm	2	3.61	**0.048**	2	3.02	*0.074*
				75th Eff Strain						
				Group	2	2.00	0.165	2	2.93	*0.079*
				Sex	1	0.00	0.968	1	2.11	0.164
				Arm	2	1.22	0.319	2	4.35	**0.029**

Cortical and trabecular effective (Eff) strain values reported for 5th, 10th, 25th, median, and 75th percentiles. *p* values represent contrast differences as assessed by Tukey’s HSD method. *p* values in bold are significant and those in italic are considered near significant, with significant contrasts detected during post hoc testing.

Within the trabecular region, group had a significant impact on 5th and 10th percentile adjusted effective strain (Table 5, Figure 2); however, no significant differences were detected between groups in the pairwise comparison. Arm had a significant impact on 75th percentile trabecular adjusted effective strain (Table 5). Ambidextrous arms (8550 ± 170 µɛ) had significantly higher 75th percentile adjusted effective strain than both dominant (7990 ± 100 µɛ, *p* = 0.025) and non-dominant (8030 ± 80 µɛ, *p* = 0.042) arms within the trabecular region.

**Figure 3:**
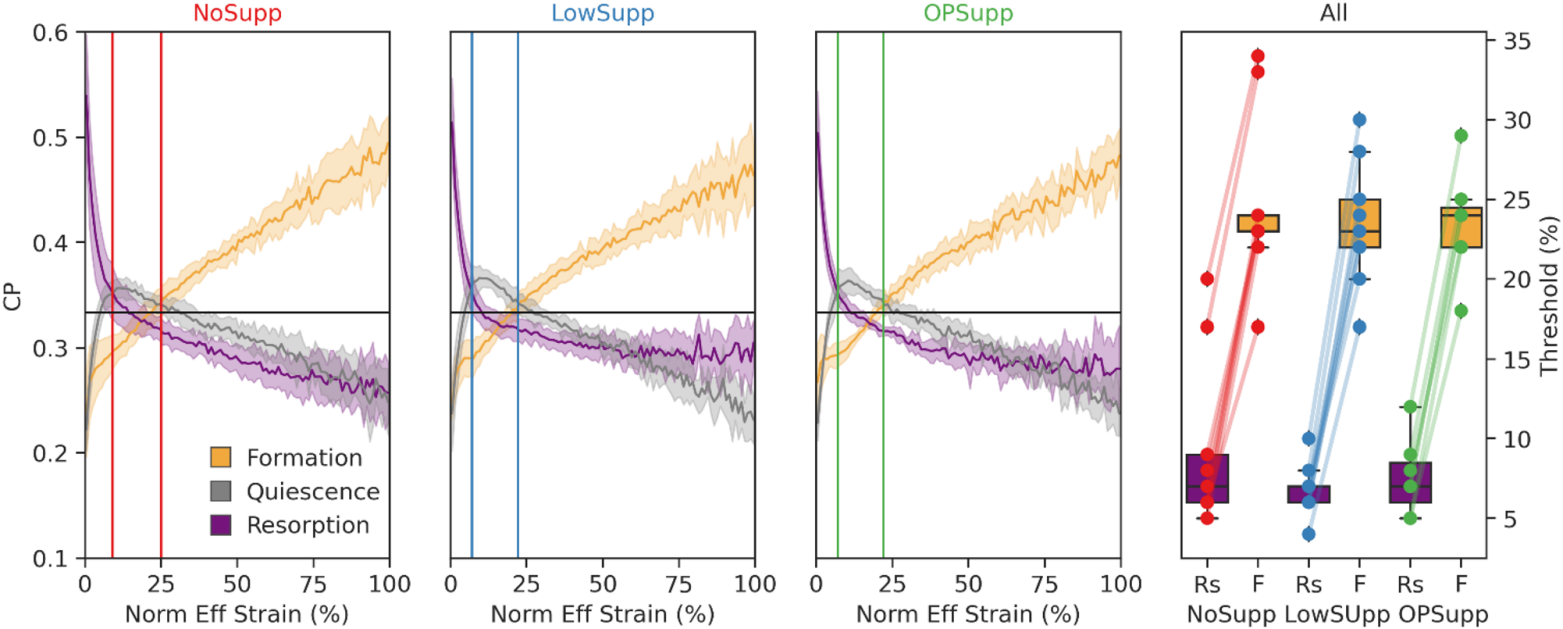
The conditional remodeling probability (CP) of remodeling sites relative to the mechanical environment, quantified as effective (Eff) strain from a simulated 1% compression, for NoSupp, LowSupp, and OPSupp groups. Normalized Eff strain distributions were used to calculate the CP for events of formation (shown in orange), quiescence (shown in grey), and resorption (shown in purple) to occur at distinct strain levels. Average thresholds dividing strains associated with resorption dominant (Rs) and formation dominant (F) probabilities each group are indicated by the left and right vertical lines, respectively, for NoSupp, LowSupp, and OPSupp (left three plots). Group and patient specific Rs and F thresholds confirmed links between bone formation at high and resorption at low mechanical signals (right).

**Figure 4:**
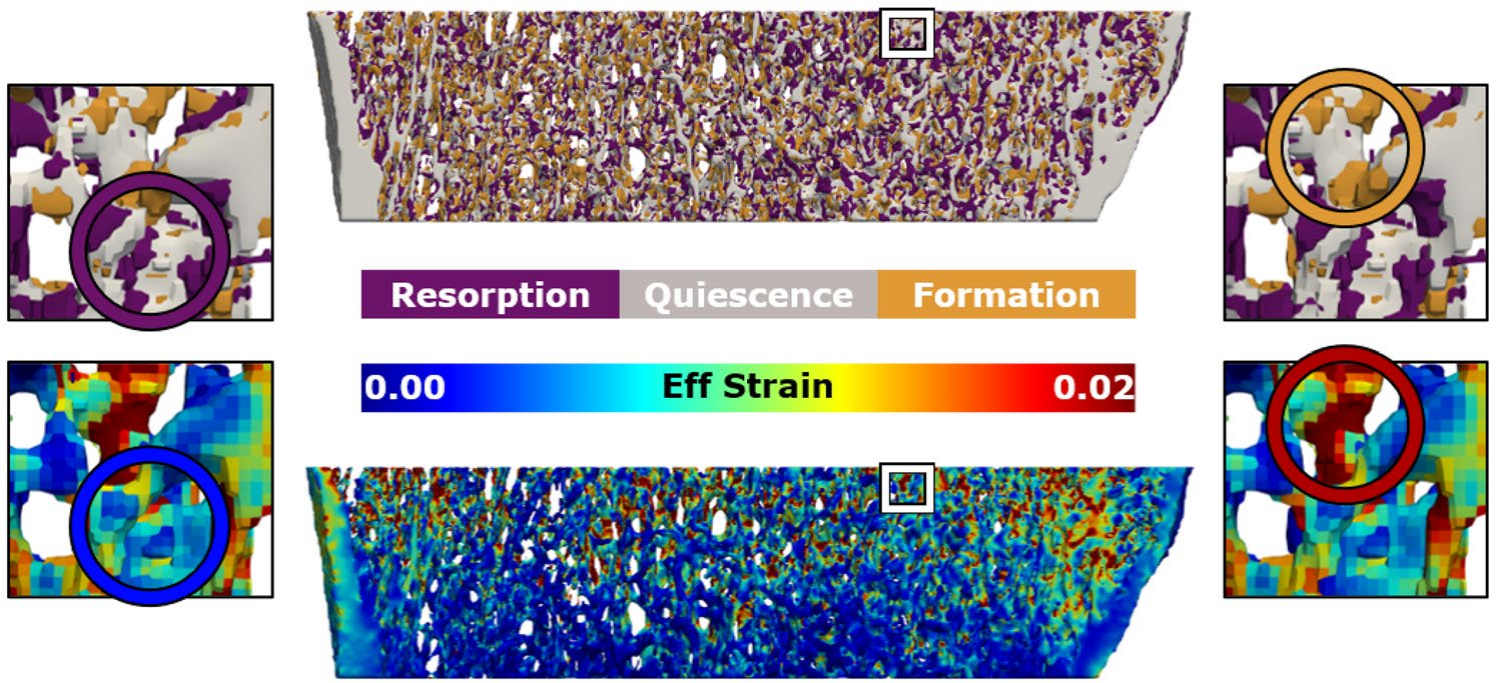
3D reconstruction of remodeling (top) and effective (Eff) strain (bottom) for a representative participant highlighting the prominence of resorption in areas of low effective strain (left) and formation in areas of higher effective strain (right).

The 99th percentile effective strain was not significantly different among groups (NoSupp: 28000 ± 2300 µɛ; LowSupp: 29200 ± 2800 µɛ; OPSupp: 26500 ± 4200 µɛ). As such, the average 99th percentile for all patients (27900 µɛ) was used to normalize the strain data from each patient for the mechanoregulation analysis. For all groups, the conditional probability (CP) of bone formation was greater at higher values of effective strain, whereas the CP of bone resorption was greater at lower values of effective strain (Figure 3). Based on these CP curves, thresholds dividing strains associated with greater probabilities of resorption and formation behavior were derived for each patient and averaged across groups. NoSupp had a lower average resorption threshold (9% strain) and higher average formation threshold (25% strain) in comparison to LowSupp and OPSupp (7% and 22% for both groups, respectively) (Figure 3). However, no significant differences in either threshold were detected among the three groups. The correct classification rate (CCR), measuring correctly classified remodeling events based on the determined resorption and formation thresholds, was similar across all groups (NoSupp = 0.408, LowSupp = 0.403, and OPSupp = 0.406) indicating consistent overall remodeling behavior. Qualitatively, regions of higher effective strain were located more distally within the analyzed region of the bone, while there were no obvious regional trends for bone remodeling (Figure 4). However, local variations in the measured mechanics and remodeling identified that lower-level regions of higher effective strain showed increased bone quality and/or quantity over the duration of the study, i.e., formation, while regions of lower effective strain showed decreased bone quality and/or quantity, i.e., resorption (Figure 4).

## Discussion

While the availability of DXA in the evaluation of patient bone mass and quantity allows for widespread use, the precision of measurement is insufficient for use in long-term patient evaluation. This study demonstrated the ability of longitudinal, extended HR-pQCT analysis to identify group-wise differences in threshold-based bone formation and resorption volume fractions (Figure 1) and mechanoregulative strategies (Figure 3). These more subtle observations in bone quality and quantity would be difficult to identify using standard clinical analyses (i.e. DXA, static morphometrics, etc.).

Generally, T-Scores were the lowest for the radius and greatest for the femur. Further, T-Scores of the radius and lumbar spine differed among the three groups, with T-Scores decreasing from NoSupp to OPSupp for both regions, as was expected based on group definitions. Although the femur is often the main target for DXA-based bone quantity scoring, as evidenced by the formulation behind the FRAX calculation for fracture risk^32^, this was the only site that showed no significant differences across groups in the current study. Regarding static morphometrics, differences were observed among groups for averaged Ct.BMD, where LowSupp had the highest value of Ct.BMD and OPSupp the lowest (Table 2). Since NoSupp had greater radius T-Scores, but lower Ct.BMD than LowSupp, this suggests there may be a compensatory increase in Ct.BMD occurring with initial reductions in areal BMD at the radius. Changes in Tb.Th observed over the duration of the study were affected by group (Table 3). Interestingly, baseline values of Tb.Th were identical for LowSupp and OPSupp; however, only LowSupp showed significantly lower adjusted mean values from NoSupp after intervention. In contrast, changes in Ct.Th observed over the duration of the study were affected by sex. Based on the magnitude of change in cortical bone morphometrics, sex appeared to have a greater impact on post-intervention values than group. Previous cross-sectional HR-pQCT studies have revealed sex-based differences in cortical bone morphometrics in both normative and pathologic patient cohorts, with males having consistently higher Ct.Th than females^24,25,33^. When comparing patients with normal bone mass and those with low bone mass and osteoporosis, significant differences in Ct.Th at the radius were detected in female patient cohorts (Normal > Low and OP)^34^, but not in male patient cohorts (Normal = Low and OP)^35^. This would indicate that sex is highly relevant to the morphometric assessment of patients for osteopenia and osteoporosis using the methods outlined herein.

Differences among the three groups were observed for cortical formation and resorption and trabecular formation. Both Ct.BMD and Ct.Th were predictors for cortical formation and resorption. LowSupp had the greatest Ct.BMD of the three groups and showed a trend for decreased resorption and formation in the cortical region, while OPSupp had the lowest Ct.BMD and showed a trend for increased formation in both the trabecular and cortical regions. Although no differences in Tb.BMD were observed, both trabecular formation and resorption were predicted by Tb.BMD and BV/TV, while trabecular resorption was also predicted by Tb.Th and the imaging interval (Table 4). In comparison to the other two groups, OPSupp showed a trend for increasing trabecular formation with increasing density, while LowSupp showed a trend for higher trabecular resorption. Combined, these results indicate that both quality and quantity drive formation and resorption volume fractions, however the specifics of this effect are difficult to quantify in this small and heterogenous cohort. Previous studies evaluating bone remodeling have not evaluated results with respect to morphometrics and densitometrics^29,30,36^, but instead found a relationship between remodeling and age^37^. However, since bone quality and quantity often decrease with age, these previous observations may indirectly support our findings.

Average stiffness values decreased with worsening T-scores, in line with previous studies linking bone loss with a drop in bone mechanical competence^10,34,35,38,39^; however, no significant differences were detected among groups after accounting for baseline stiffness. Further, none of the investigated covariates had a significant influence on post-intervention stiffness. Herein, the interval between baseline and follow-up may have been too short to detect differences in the mechanics at the organ-level. A review assessing the clinical application of HR-pQCT in adult patient populations found less than half of studies assessing bone strength (i.e. stiffness and failure load) reported significant differences between anti-osteoporotic drug treatment and placebo groups, with the majority of trials running for more than 12 months^26^. Of the studies that had a 12 month follow-up interval, only one reported significant changes in response to treatment^40^. Combined with the results of the current work, this indicates the need to establish guidelines for minimum follow-up intervals in longitudinal HR-pQCT studies.

Few studies have explored changes in *in vivo* strain distribution in longitudinal analysis of human bone, often focusing on regional median or average values^21,30,41^. Although not directly comparable, the patterns of strain distribution in the cortical and trabecular regions from this study are consistent with Johnson and Troy^42^. The bulk of trabecular strains were lower than those in the cortical region; however, peak strains were measured in the trabecular region, likely because of thin individual trabeculae. The use of a high-friction compression boundary condition may have also oversimplified the true in vivo loading environment, contributing to differences in the pattern of strain distribution at or near the edges of the model. Despite this, consistent patterns of load transfer from the trabecular compartment to the cortical shell were observed in all models. Moreover, the high-friction compression boundary condition is most used in the evaluation of HR-pQCT-based FE models, enabling greater cross-comparability to existing and future studies. At the tissue level, group differences were detected in both cortical and trabecular mechanical properties. Within the cortical region, the adjusted strain distribution (10th, 25th, and 50th percentiles) was lower for LowSupp compared to OPSupp following intervention. Within the trabecular region, group differences were detected in the lowest (5th and 10th percentile) adjusted strain values; however, no significant differences were detected between groups in the post hoc analysis. Although the detected differences were small (3–5%) this could be reflective of small changes in the mineralization at the voxel level that were not detected in the morphological analysis. Since the material properties were derived directly from the voxel intensity, minute, local increases in density could result in a stiffening of the bone material within the model. Given that the boundary conditions were constant, changes in material stiffness would result in a drop in the measured strain.

Previous imaging studies have found significantly greater macrostructural and mechanical properties in the dominant radius compared to the non-dominant radius^43,44^. Specifically, bone area and BMC from pQCT-based (330 µm voxels) studies and cortical area and failure load from HR-pQCT-based (82 µm voxels) studies were higher in dominant radii. More recently, studies using second generation HR-pQCT (60.7 µm voxels) to study arm dominance have also reported significantly increased macro- and microstructure and mechanical properties in the dominant arm^45,46^. Only one study has reported ambidextrous or equivalent arm dominance, but only one participant identified as such^46^. In the current study, arm dominance had no impact on post-intervention density or morphological parameters. Regarding post-intervention mechanics, ambidextrous arms were found to have significantly higher adjusted strains than the non-dominant (cortical: 10th. 25th, and 50th percentile strain; trabecular: 75th percentile) and dominant arms (cortical: 25th percentile strain; trabecular: 75th percentile strain). No differences in post-intervention response were detected between dominant and non-dominant arms. Given that the participants in the current study suffered a fracture on the non-investigated arm, the observed differences may have resulted due to changes in the daily loading pattern or usage of the contralateral arm. Participants with dominant arm fractures (non-dominant contralateral arms) likely experienced the greatest overall effect to activity and ability as they may not have been proficient in use of their contralateral arm, while participants with non-dominant arm fractures (dominant contralateral arms) or who were ambidextrous experienced little effect to their activity of daily living. Therefore, patients with ambidextrous arm dominance may have experienced the greatest increase in contralateral arm activity, due to their existing proficiency and increase in daily use. This increase in stimulus may explain our results; however, as only three participants identified as having ambidextrous arm dominance, this observed effect requires further investigation for confirmation.

Consistent with previous studies which found normal physiological activity levels result in significant relationships between bone formation and FE-derived mechanical stimulus^30,31^, the mechanoregulation analysis revealed strong relationships between local mechanics and remodeling in all groups. Trends were observed in the magnitude of the difference between the resorption and formation threshold values (i.e. width of the lazy zone^30^) and the relative position of this zone, among both groups and individuals. Qualitatively, NoSupp had the widest lazy zone, while LowSupp and OPSupp had narrower lazy zones with thresholds shifted towards lower effective strain values (Figure 3). Observation of this trend suggests patients in LowSupp and OPSupp were more reactive to stimuli (or the lack thereof), requiring less mechanical signal to prompt bone remodeling than those in NoSupp, but requires a larger, and potentially more homogeneous, cohort to confirm this finding. Although age was not a significant factor, the two youngest participants in the current study were in the NoSupp group and both had higher thresholds for formation and resorption as well as a wider lazy zone than the other participants. Future studies should explore the capabilities of HR-pQCT-based mechanoregulation analysis for addressing differences in participant activity level and age.

This study does have limitations. First, the evaluated participant cohorts were small in number and were grouped based on a combination of bone density measurements from DXA and prescription of supplements based on blood biomarkers, thus the groups do not allow for direct translation to the effect of either initial bone quality and quantity or supplements, as these factors were not analyzed independently. Further, due to limited availability of patient history, the degree to which patients adhered to their prescribed supplements is unknown. Additionally, potentially relevant clinical factors (fall history, activity level, etc.) were not able to be included in this evaluation. However, even with a relatively small cohort of patients, this study observed variability in remodeling and mechanoregulation among the groups, which indicates that the sensitivity of HR-pQCT should be further investigated in the clinical evaluation of patients. Second, remodeling and mechanoregulation of the contralateral arm may not be independent of the healing process of the fractured arm. As such, results may have been influenced by the severity of the fracture and change in the dependence on the contralateral arm during healing, as this would vary depending on whether the fractured arm was dominant or not. To address this, dominance of the evaluated arm was included as a factor in our analysis in order to separate this factor from the observed results and was found to affect low to median cortical effective strain.

## Conclusion/outlook

Longitudinal HR-pQCT was able to detect differences between our three cohorts in threshold-based formation and resorption volume fractions and the factors driving mechanoregulation over periods of 9–12 months. While our study sample size limits our ability to identify population-based findings, our results indicate that extended HR-pQCT analyses are able to detect subtle differences in remodeling and mechanoregulation strategies that may be indicative of bone quality and quantity. Thus, when the imaging technology is available, clinicians should consider supplementing current patient evaluation protocols with time-lapsed microstructural imaging and analysis. Additional research is necessary to highlight the specific clinical benefits, however the use of longitudinal, extended HR-pQCT shows promise in improving future diagnosis and treatment strategies to drive patient-specific plan of care for bone health.

## Methods

A subset of 25 subjects who were recruited and gave informed consent for their participation in a time-lapse HR-pQCT imaging study were analyzed herein (Table 1). All experimental protocols were approved by the Ethics Committee of the Medical University of Innsbruck (UN 0374344/4.31) and carried out according to the Declaration of Helsinki. Blood samples (35 ml) were analyzed to assess calcium, 25-hydroxyvitamin D [25(OH)D], and parathyroid hormone (PTH) in the Medical University Laboratory, Innsbruck, where values were considered normal in the ranges of 2.20–2.55 mmol/l for calcium, 75–150 nmol/l for 25(OH)D, and 15–65ng/l for PTH and the decision for prescription of supplements was made by the treating doctor. For patients with low values of the general bone markers, vitamin D3 (5 subjects) or a combination of vitamin D3 and calcium (11 subjects) supplements were recommended and patient compliance was verified. In isolation, the prescribed dose of vitamin D3 was ~ 8000 IU daily (Oleovit D3, 10 to 20 drops/day) for two-weeks followed by ~ 1000 IU daily (Oleovit D3, 15 to 25 drops/week) for the remainder of the study. The prescribed dose for combined supplementation consisted of 400 to ~ 1000 IU vitamin D3 and 500–600 mg calcium daily (Maxi-Kalz, 500 mg calcium, and Oleovit, ~ 1000 IU vitamin D3; Calciduran, 500 mg calcium and 800 IU vitamin D3; or Cal-D-Vita, 600 mg calcium and 400 IU vitamin D3). All subjects were above 18 years of age, had a unilateral distal radius fracture, and provided informed consent prior to their participation. To eliminate the convoluting effect of fracture healing on bone remodeling and mechanoregulation, only images of the contralateral, non-fractured radius were analyzed.

### Image acquisition and clinical metrics

Data was obtained as part of an unrelated study investigating fracture healing. HR-pQCT (XtremeCT II, Scanco Medical AG, Brütisellen, Switzerland) images (168 slices, 10.2 mm scan length, 60.7 μm isotropic voxels, 63 kV, 1500 µA, 46 ms integration time, 2304 samples, 900 projections) of the contralateral radius were acquired at six time points during the first-year post-fracture (approximately 1, 3, 5, 13, 26, and 52 weeks post-fracture). The standard clinical evaluation was completed at Innsbruck Medical University which provided both densitometric indices, including volumetric bone mineral density (BMD) for the whole bone (Tt.BMD), trabecular (Tb.BMD), and cortical (Ct.BMD) regions, and morphometric indices, including bone volume fraction (BV/TV), trabecular number (Tb.N), trabecular separation (Tb.Sp), mean thickness of the trabecular (Tb.Th) and cortical (Ct.Th) regions, and cortical porosity (Ct.Po) of each study participant. Dual-energy X-ray absorptiometry (DXA) images were acquired three-weeks post fracture for the femur, lumbar spine and radius and were used to quantify the T-score of each subject. Only participants with at least two high quality images of the contralateral radius (visual grading score, VGS^47^, of 1 or 2) taken 9–12 months apart were considered for inclusion in this study.

### Dynamic morphometry

The two images which had the best image quality (lower VGS)^47^ and greatest volume of overlap were used to assess bone formation, resorption, and quiescence. As previously described^29^, the earlier of the two images was transformed using cubic interpolation to be aligned with the imaging coordinate system using the SciPy function library in Python^48,49^. The later of the two images was then rigidly registered and transformed to align with the earlier image using a pyramid-based approach optimized relative to the mean squared error between the two images^50^. Masks of the radius in each image were generated using geodesic active contouring^51^ and used to generate cortical and trabecular masks using the scanner manufacturer’s software.

Images were de-noised with a constrained Gaussian filter (sigma = 1.2, truncate = 0.8, support = 1.0) in Python and thresholds ranging from 200 to 920 mg Hydroxyapatite (HA)/cm^3^ were applied with 120 mg HA/cm^3^ intervals. Note, thresholds greater than 680 mg HA/cm^3^ were ignored in the trabecular bone based on the lower mineralized density of trabecular bone and minimal trabecular bone volume measured at higher densities. A threshold of 320 mg HA/cm^3^ was defined as a standardized threshold for analysis, based on the use as a trabecular threshold in previous studies^52^. The two images were then compared to determine voxels that had formed, resorbed, or were quiescent at each mineralized density threshold^53^. Formation and resorption volume fractions were calculated relative to the bone volume at each threshold from the earlier image.

### Computational mechanics

The overlapping, registered HR-pQCT data were used to generate two micro-FE models for each patient via direct conversion of the image voxels to hexahedral elements (Python 3.7). Scaled, linear elastic material properties, computed directly from the Gaussian filtered (sigma = 1.2, truncate = 0.8, support = 1.0) density data using SciPy^48^, and a Poisson’s ratio of 0.3 were assigned to all elements. High friction compression tests with a prescribed 1% displacement of the total height in the axial direction were performed on all models using 180 CPUs from a CRAY XC40 (Swiss National Supercomputing Centre (CSCS)). Results from models were used to calculate apparent compressive stiffness of the contralateral radii over time and to evaluate longitudinal changes in the effective strain (ε_Eff_) distribution within cortical and trabecular bone. Effective strain, a scalar strain measure, was calculated from the strain-energy density (SED) and the Young’s modulus of the bone tissue (E), calculated directly from the density of each voxel within the region of the bone using equation (1)^53,54^. The full-field strain data in each bone compartment was sampled at the 5th, 10th, 25th, 50th, and 75th percentiles to enable quantitative comparisons among the NoSupp, LowSupp, and OPSupp groups over the course of the study. Low, median, and relatively high strain percentiles were sampled to better characterize the shape of the strain distributions in lieu of only reporting the median or average ε_Eff_.

1 $${\varepsilon }_{eff}=\sqrt{\frac{2*SED}{E}}$$

Results from the FE analyses were spatially correlated with formation, resorption and quiescent bone volumes to assess local mechanoregulation. Here, conditional probability (CP) curves were generated for the remodeling events identified on the bone surface^55,56^, connecting the local mechanical environment (ε_Eff_) with the observed formation, resorption or quiescence events. The effective strain distribution for each FE analysis was normalized using the average 99th percentile of the whole cohort and binned at 1% steps for each remodeling event. A group- and bin-wise normalization were used to calculate CP curves for each subject group in accordance with Schulte et al., 2013^55^. A correct classification rate (CCR), measuring the fraction of correctly identified remodeling events using the CP curves^53^, was calculated to summarize mechanoregulation within each group. Additionally, formation (Tf) and resorption (Tr) strain thresholds were derived from the CP curves for each subject at the point for which formation or resorption became dominant, respectively.

### Statistical analysis

The Python SciPy function library was used to report and evaluate differences in formation and resorption bone volume fractions and mechanics^48^. The average and difference of densitometry and morphometry measures were used for analysis of each group. Normality of data was evaluated using the Shapiro-Wilk test for normality. Group differences were investigated using Kruskal-Wallis one-way analysis of variance (ANOVA) on ranks when data was non-normally distributed.

To determine the effect of treatment on the morphometrics and mechanics of each group, an analysis of covariance (ANCOVA) was performed in R (R version 4.0.4). Here, baseline measurements, group, imaging interval, age, sex, and dominance of the evaluated arm were included as covariates to predict post-intervention measurements^57,58^. Imaging interval and age were treated as continuous variables, while group (3 levels: [0] NoSupp, [1] LowSupp, [2] OPSupp), sex (2 levels: [0] female, [1] male), and arm (3 levels: [0] ambidextrous, [1] dominant, [2] non-dominant) were categorical variables. Pairwise comparisons were performed for all covariates that had a significant (*p* < 0.05) and near significant (*p* < 0.1) effect on post-intervention values. Tukey’s HSD method was applied to account for multiple comparisons and resulting values are represented as adjusted means ± standard error. Post-hoc analysis was completed using Dunn's test with Bonferroni correction or Tukey-Kramer when family-wise error was present.

To investigate the parameters which had the largest effect on formation and resorption volume fractions, partial least squares (PLS) regression was performed on each volume fraction including variables of demographics, group, densitometry and morphometry data, DXA-measured T-scores, and density threshold using the Python Scikit-Learn function library^59^. All variables were scaled and centered prior to analysis. Leave-one-out cross-validation was used to calculate the predictive power of the model and the number of model components was limited to one. The variables were sorted by variable influence on projection (VIP) and the model was run iteratively including additional variables until the Q^2^ score, which is a measure of predictability equivalent to an R^2^ value, no longer improved. Root mean squared error of the prediction (RMSEP) was included as an additional measure of the regression. Due to our desire to explore the capabilities of enhanced, longitudinal HR-pQCT, we did not restrict the number of variables chosen in the analysis based on the limited size of our three groups.

## Data Availability

The datasets analyzed within the current study are available from the corresponding author upon reasonable request.
